# Applications of natural language processing at emergency department triage: A narrative review

**DOI:** 10.1371/journal.pone.0279953

**Published:** 2023-12-14

**Authors:** Jonathon Stewart, Juan Lu, Adrian Goudie, Glenn Arendts, Shiv Akarsh Meka, Sam Freeman, Katie Walker, Peter Sprivulis, Frank Sanfilippo, Mohammed Bennamoun, Girish Dwivedi

**Affiliations:** 1 School of Medicine, The University of Western Australia, Crawley, Western Australia, Australia; 2 Harry Perkins Institute of Medical Research, Murdoch, Western Australia, Australia; 3 Department of Emergency Medicine, Fiona Stanley Hospital, Murdoch, Western Australia, Australia; 4 Department of Computer Science and Software Engineering, The University of Western Australia, Crawley, Western Australia, Australia; 5 HIVE & Data and Digital Innovation, Royal Perth Hospital, Perth, Western Australia, Australia; 6 Department of Emergency Medicine, St Vincent’s Hospital Melbourne, Melbourne, Victoria, Australia; 7 SensiLab, Monash University, Melbourne, Victoria, Australia; 8 School of Clinical Sciences at Monash Health, Monash University, Melbourne, Victoria, Australia; 9 Western Australia Department of Health, East Perth, Western Australia, Australia; 10 School of Population and Global Health, University of Western Australia, Crawley, Western Australia, Australia; 11 Department of Cardiology, Fiona Stanley Hospital, Murdoch, Western Australia, Australia; Deakin University, AUSTRALIA

## Abstract

**Introduction:**

Natural language processing (NLP) uses various computational methods to analyse and understand human language, and has been applied to data acquired at Emergency Department (ED) triage to predict various outcomes. The objective of this scoping review is to evaluate how NLP has been applied to data acquired at ED triage, assess if NLP based models outperform humans or current risk stratification techniques when predicting outcomes, and assess if incorporating free-text improve predictive performance of models when compared to predictive models that use only structured data.

**Methods:**

All English language peer-reviewed research that applied an NLP technique to free-text obtained at ED triage was eligible for inclusion. We excluded studies focusing solely on disease surveillance, and studies that used information obtained after triage. We searched the electronic databases MEDLINE, Embase, Cochrane Database of Systematic Reviews, Web of Science, and Scopus for medical subject headings and text keywords related to NLP and triage. Databases were last searched on 01/01/2022. Risk of bias in studies was assessed using the Prediction model Risk of Bias Assessment Tool (PROBAST). Due to the high level of heterogeneity between studies and high risk of bias, a metanalysis was not conducted. Instead, a narrative synthesis is provided.

**Results:**

In total, 3730 studies were screened, and 20 studies were included. The population size varied greatly between studies ranging from 1.8 million patients to 598 triage notes. The most common outcomes assessed were prediction of triage score, prediction of admission, and prediction of critical illness. NLP models achieved high accuracy in predicting need for admission, triage score, critical illness, and mapping free-text chief complaints to structured fields. Incorporating both structured data and free-text data improved results when compared to models that used only structured data. However, the majority of studies (80%) were assessed to have a high risk of bias, and only one study reported the deployment of an NLP model into clinical practice.

**Conclusion:**

Unstructured free-text triage notes have been used by NLP models to predict clinically relevant outcomes. However, the majority of studies have a high risk of bias, most research is retrospective, and there are few examples of implementation into clinical practice. Future work is needed to prospectively assess if applying NLP to data acquired at ED triage improves ED outcomes when compared to usual clinical practice.

## Introduction

Millions of patients attend emergency departments (EDs) around the world every year [1]. Queues for care are common, so patients are often triaged on arrival to the ED by a trained nurse. Triage is central to the practice of emergency medicine [2]. In the face of excess demand, triage allows EDs to allocate their finite resources in an equitable, efficient, and standardised way [3, 4]. Triage systems in current use include the Emergency Severity Index (ESI), Australasian Triage Scale (ATS), Manchester Triage Scale (MTS), and the Korean Triage and Acuity Scale (KTAS) [3, 5]. Triage systems aim to aid emergency care providers in making a structured decision regarding the urgency of care that a patient requires, and in doing so, identify and prioritise those patients with time-sensitive care needs [3, 4]. No triage tool is perfect, and all have issues with sensitivity and specificity resulting in over and under-triage, particularly for certain demographic groups and conditions [6–8]. There is opportunity to improve triage performance in identifying patients with critical illness, and for improving triage accuracy and the consistency of triage categorisation between healthcare workers [3].

Machine learning (ML) is a subfield of artificial intelligence (AI), that uses various methods to automatically deduce patterns in data, then make predictions [9]. These patterns are learned from the data rather than being explicitly pre-programmed by humans. ML models are iteratively improved through a process called training. In supervised ML training, the model’s predicted output is compared to a "ground truth", and the error between the predicted value and ground truth is progressively reduced through the training process [9]. ML models may have the potential to improve risk stratification and outcome prediction in the ED setting [10–12].

Triage has been identified as a promising area to apply ML in the ED [13, 14]. ML has previously been applied to structured data acquired at triage (such as patient age and vital signs) in attempts to predict outcomes including need for admission and intensive care [15, 16]. Triage nurses routinely collect structured data and an unstructured free-text history of presenting complaint, capturing their impression and subjective assessment about the presentation. This free-text may be more expressive, nuanced, and contain a higher level of information than structured data [17]. Prior work has suggested that incorporating free-text may improve the performance of ML at ED triage and is an important area for future research despite the challenges of incorporating free-text data into models [18–20].

Natural language processing (NLP) uses computational methods to analyse and understand human language and its structure [21]. Early NLP techniques were relatively simple. For example, a “bag-of-words” model bases its decision on the relative frequencies of words in the text, ignoring their order [22]. These early models often lacked the ability to assess context, negations, and as a result had numerous limitations [23]. Significant advancements in NLP have been made over the last few years through the use of Deep Learning (DL), a subfield of ML [24, 25]. DL models pass data through multiple processing layers and in doing so, achieve increasingly abstract representations of the input data, enabling them to learn complex functions [26]. Massive DL based NLP models have recently been developed [27–29]. These models have been trained on datasets containing billions of words and have achieved high levels of performance [27–29]. Some large, pre-trained models, such as Bidirectional Encoder Representations from Transformers (BERT) are publicly available [27]. Using a pre-trained model allows researchers to take a high performing model as their starting point, and then customise it to their unique needs through fine tuning the model on their local data. For example, Tahayori et al. were able to accurately predict admission from ED using only free-text triage notes and a BERT based NLP model [30]. Multimodal models integrate NLP with other types of ML to analyse combinations of both free-text data and structured data (such as age and vital signs).

### Objectives

This review aims to evaluate the applications of NLP at ED triage by answering the following questions:

1. How has NLP been applied to data acquired at ED triage?
2. Do NLP based models outperform humans or current risk stratification techniques when predicting outcomes?
3. Does incorporating free-text improve predictive performance of ML models when compared to ML models that use only structured data?

## Methods

A review protocol was prepared in accordance with PRISMA-P guidelines and registered with the International Prospective Register of Systematic Reviews (PROSPERO) on 04/10/2021 (Registration ID: CRD42021276980) [31, 32]. All English language peer-reviewed research that applied an NLP technique to free-text obtained at ED triage were eligible for inclusion. As this study aims to broadly assess the capability of NLP at triage, all outcomes and comparators were included. We excluded studies focusing solely on disease surveillance, and studies that used information obtained after triage (such as emergency physician clinical notes and investigations performed within the ED).

We searched PubMed (MEDLINE), Embase, Cochrane Database of Systematic Reviews, Web of Science, and Scopus for research published from database inception to the present day. Electronic databases were first searched on 16/09/2021 and last searched on 01/01/2022. We searched for medical subject headings (MeSH) and text keywords related to NLP and triage. The search strategy was iteratively developed by the multidisciplinary project team that included emergency physicians and computer scientists (S1 File). Reference lists of the included studies and the authors’ personal archives were reviewed for further relevant literature.

Citations and abstracts were screened independently by two reviewers (JS and JL) against the inclusion and exclusion criteria. Both reviewers were blind to the journal titles, study authors, and institutions. Full text articles were obtained for any articles identified by one reviewer to meet inclusion criteria. Two reviewers (JS and JL) then evaluated the full text reports against the inclusion and exclusion criteria. Data were extracted by JS and JL using a standardised form that included study country, study design, outcomes, number of sites, study population, input data, NLP and ML models used, comparison, and results. The form was piloted, and calibration exercises were conducted prior to formal data extraction to ensure consistency between reviewers. In cases of conflict or discrepancy, additional review authors were involved until a decision was reached. There were no uncertainties that required authors of the included studies to be contacted. Risk of bias in studies was assessed independently by two authors (JS and JL) using the Prediction model Risk of Bias Assessment Tool (PROBAST) [33]. We chose PROBAST as it is a well-designed, commonly used, and generally accepted tool to assess risk of bias and applicability concerns in prediction model studies.

Heterogeneity was assessed by the study team through review of included papers and the results table. The application of NLP to data acquired at triage was not a homogenous and consistent intervention. Initial review of included studies revealed a wide range of settings with associated differences in language, healthcare systems and triage systems. Further variation is seen in the range of inputs and then these inputs were then studied using different models based on a wide variety of ML approaches (ranging from traditional ML techniques to early deep learning models to modern transformer-based techniques). The outputs for the models are also variable including disposition, identification of critical illness, triage score, investigation ordering and specific disease groups. Most studies were subsequently assessed as having a high risk of bias. Despite some studies assessing NLP performance in predicting similar outcomes, models and inputs used were often very different. There were no randomised controlled studies, few studies compared NLP to usual practice, and few studies had an appropriate comparator. Due to these reasons, the consensus decision by the research team is that a meta-analysis would not be meaningful, and would likely be misleading. Instead, a narrative synthesis is provided to summarise review findings.

## Results

### Study selection

**Fig 1:**
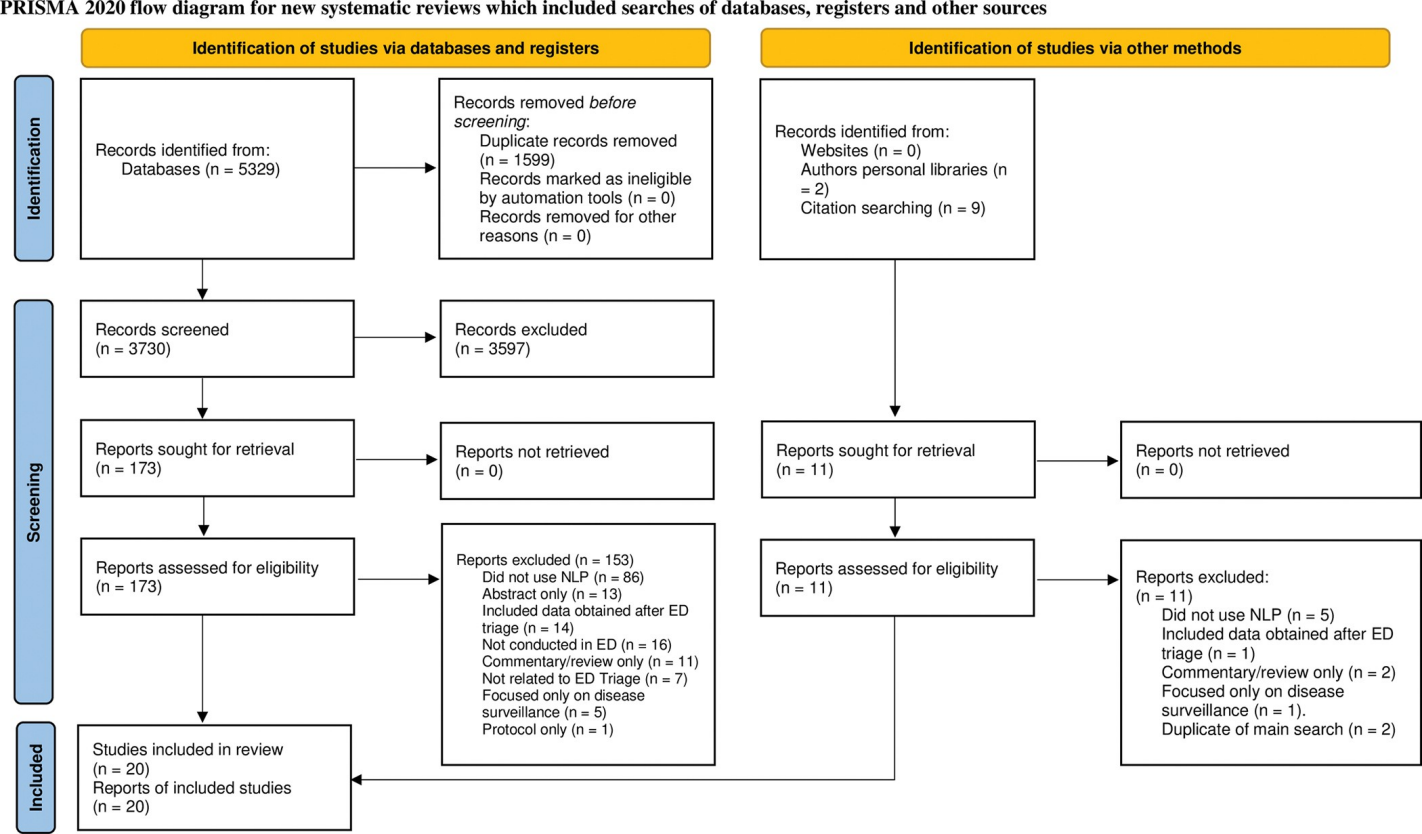
Prisma flow diagram.

This process is summarised in a PRISMA Flow Diagram (Fig 1). There were 5329 records identified following database searching and a further 11 records identified through other sources. Following removal of duplicates, 3730 records remained and underwent title and abstract screening. 3597 records were excluded. The remaining 173 full-text articles were assessed for eligibility. In total, 153 articles were excluded, and 20 studies remained for inclusion (Fig 1). There were no unresolved disagreements as to study inclusion or results of data extraction.

### Characteristics of included studies

A summary of the included studies is shown in Table 1. There were 19 retrospective studies [17, 18, 30, 34–49]. One study reported their ML model was developed using retrospective data then validated using prospective data [50]. All used observational cohort designs. Two studies were international multi-centre studies (USA and Portugal); 12 were conducted in the USA; 2 were from South Korea; one each from Australia, Brazil, China, and France. The most common outcomes assessed were prediction of triage score (six studies), prediction of admission (five studies), and prediction of critical illness (three studies). Two studies predicted need for imaging within the ED, two studies looked at the assignment of provider assigned chief complaint label, one study predicted diagnosis of infection in the ED, and one study aimed to identify and classify temporal expressions used in triage notes.

**Table 1:** Details of included studies.

Study	Country	Study design	Outcome	Sites	Population	Input data
Kim et al. (2021)	South Korea	Retrospective	Assignment of triage score (KTAS).	Single-centre (1 site)	762 simulated triage cases	Human transcribed and ML-transcribed simulated triage dialogue
Ivanov et al. (2021)	USA	Retrospective	Assignment of triage score (ESI).	Multi-centre (2 sites)	147 052 patient encounters (age 1 year or older)	Blood glucose, chief complaint (free-text), demographics, FHx, history of presenting complaint (free-text), mental status, mode of arrival, pain score, SHx, vitals
Tahayori et al. (2020)	Australia	Retrospective	Patient disposition (admission or discharge).	Single-centre (1 site)	249 532 patient encounters (adult)	History of presenting complaint (free-text)
Sterling et al. (2020)	USA	Retrospective	Assignment of triage score (ESI).	Multi-centre (3 sites)	226 317 patient encounters (adult and paediatric)	Chief complaint (structured), demographics, history of presenting complaint (free-text), medication, mental status, mode of arrival, pain score, PMHx, vitals
Roquette et al. (2020)	Brazil	Retrospective	Patient disposition (admission or discharge).	Single-centre (1 site)	499 853 patient encounters (paediatric)	Blood glucose, chief complaint (free-text), demographics, history of presenting complaint (free-text), medication, pain score, past investigation requests, PMHx, triage score (MTS), vitals
Joseph et al. (2020)	USA	Retrospective	Identification of critical illness (death within 24 hours of arrival, ICU admission from the ED or within 24 hours of ward admission).	Single-centre (1 site)	445 925 patient encounters (adult)	Demographics, chief complaint (free-text), triage score (ESI), vitals
Fernandes et al. (2020) (1)	Portugal, USA	Retrospective	Identification of critical illness (ICU admission within 24 hours of triage).	Multi-centre (2 sites)	Site one 120 649 patient encounters (adult) Site two 235 826 patient encounters (adult)	Blood glucose, chief complaint (structured and free-text), exams prescribed at triage, mental status, mode of arrival, pain score, time of triage, triage score (ESI or MTS), vitals
Fernandes et al. (2020) (2)	Portugal, USA	Retrospective	Identification of critical illness (in-hospital death or cardiopulmonary arrest within 24 hours of triage).	Single-centre (1 site)	235 826 patient encounters (adult)	Blood glucose, chief complaint (free-text), exams prescribed at triage, mental status, mode of arrival, pain scale, time of triage, vitals
Chang et al. (2020)	USA	Retrospective	Prediction of provider-assigned chief complaint label.	Multi-centre (7 sites)	1 799 365 free-text chief complaints (adult and paediatric)	History of presenting complaint (free-text)
Arnaud et al. (2020)	France	Retrospective	Patient disposition (admission or discharge).	Single-centre (1 site)	Approximately 190 000 patient encounters (adult)	Arrival time, bladder volume, blood glucose, breath alcohol, capillary haemoglobin, capillary ketones, demographics, history of presenting complaint (free-text), mode of arrival, pain, PMHx (free-text), triage score, vitals
Zhang et al. (2019) (1)	USA	Retrospective	Use of advanced diagnostic imaging (CT, US, MRI) during ED visit.	Multi-centre (300 sites)	139 150 presentations (adult)	Arrival time, demographics, history of presenting complaint (free-text), mode of arrival, pain scale, PMHx, triage score, vitals, whether the visit was related to an injury/poisoning/adverse effect of medical treatment
Zhang et al. (2019) (2)	USA	Retrospective	Performance of any diagnostic imaging during ED visit.	Multi-centre (300 sites)	27 665 patient encounters (paediatric)	Arrival time, demographics, history of presenting complaint (free-text), mode of arrival, pain scale, PMHx, triage score, vitals, whether the visit was related to an injury/poisoning/adverse effect of medical treatment
Wang et al. (2019)	China	Retrospective	Assignment of triage score.	Single-centre (1 site)	70 918 patient encounters (adult)	Chief complaint (free-text), demographics, history of presenting complaint (free-text), physical examination (free-text), vitals
Sterling et al. (2019)	USA	Retrospective	Patient disposition (admission or discharge).	Multi-centre (3 sites)	256 878 patient encounters.	History of presenting complaint (free-text)
Greenbaum et al. (2019)	USA	Retrospective then prospective	Percent of presenting problems entered at triage able to be automatically mapped to a structured ontology.	Single-centre (1 site)	279 231 patient encounters total (78 157 patient encounters were post-implementation)	Demographics, history of presenting complaint (free-text), pain score, triage score (ESI), vitals
Choi et al. (2019)	South Korea	Retrospective	Assignment of triage score (KTAS).	Single-centre (1 site)	138 022 patient encounters (adults)	Arrival time, chief complaint (structured), demographics, history of presenting complaint (free-text), mental status, mode of arrival, pain location and intensity, vitals
Gligorijevic et al. (2018)	USA	Retrospective	Assignment of triage score (ESI).	Single-centre (1 site)	338 500 patient encounters	Arrival time, chief complaint (free-text), demographics, history of presenting complaint (free-text), medication, mode of arrival, PMHx, vitals
Zhang et al. (2017)	USA	Retrospective	Patient disposition (admission or discharge).	Multi-centre (642 sites)	47 200 patient encounters (paediatric and adult)	Arrival time, demographics, chief complaint (free-text), mode of arrival, pain score, PMHx, triage score, vitals, whether the visit was related to an injury/poisoning/adverse effect of medical treatment
Horng et al. (2017)	USA	Retrospective	Diagnosis of infection in the emergency department.	Single-centre (1 site)	230 936 patient encounters	Demographics, chief complaint (free-text), history of presenting complaint (free-text), pain score, triage score (ESI), vitals
Irvine et al. (2008)	USA	Retrospective	Identification and classification of temporal expressions used in triage notes.	Multi-centre (94 sites)	598 triage notes	Arrival time, History of presenting complaint (free-text)

Abbreviations

KTAS—Korean Triage and Acuity Scale

ESI—Emergency Severity Index

ICU—Intensive Care Unit

ED—Emergency Department

ML—Machine learning

FHx—Family history

SHx—Social history

PMHx—Past medical history

Vitals—Respiratory rate (RR), heart rate (HR), systolic blood pressure (SBP), diastolic blood pressure (DBP), temperature (Temp), and oxygen saturation (SPO2).

MTS—Manchester Triage system

The population size varied greatly between studies ranging from 598 triage notes to 1.8 million patients. Five studies used a population of under 100 000, four studies had a population of between 100 000 and 200 000, six studies had a population of between 200 001 and 300 000, and six studies had a population of over 300 000. Twelve studies used data from a single site and eight studies used data from multiple sites. The largest number of sites used was 642 by Zhang et al.

Fifteen studies applied NLP to free-text history of presenting complaint, seven studies applied NLP to a free-text chief complaint, two studies applied NLP to a structured chief complaint label, and one study applied NLP to simulated triage dialogues that had been transcribed by either a human or an ML model. The other most frequently used input variables were patient demographics (13 studies), patient vital signs (heart rate, respiratory rate, oxygen saturation, blood pressure, and temperature) (15 studies), pain score (12 studies), triage score (10 studies), mode of arrival (10 studies), time of arrival (9 studies) and past medical history (7 studies). Other input variables included mental status (5 studies), and blood glucose level (5 studies).

### Prediction of admission

Overall, NLP models and multimodal models achieved high Area Under the Receiver Operating Characteristic Curve (AUC) in predicting admission at time of triage for adult and paediatric patients (Table 2) [18, 30, 35, 41, 46]. Of the five studies focusing on predicting admission to hospital, Roquette et al. achieved the highest (AUC) using a gradient boosting model (AUC 0.89). Tahayori et al. achieved a similar AUC (0.88) using only free-text history of presenting complaint. Tahayori et al. were the only authors that compared their model to emergency physician performance. Their model achieved a higher accuracy than five emergency consultants (0.83 vs 0.78) and higher specificity (0.86 vs 0.77), but lower sensitivity (0.72 vs 0.9). Roquette et al. and Zhang et al. both compared ML models trained using structured data only with ML models that incorporated both structured data and text data. They found that the addition of text data results in a small improvement (Zhang AUC 0.823 to AUC 0.844, Roquette AUC 0.872 to AUC 0.891) when compared to the use of structured data alone. This improvement was not assessed for statistical significance.

**Table 2:** Results of included studies, grouped by outcome.

Study	Outcome	Population	Dataset characteristics	Input data	NLP/ML Model	Comparison	Results
Kim et al. (2021)	Assignment of triage score (KTAS).	762 simulated triage cases	KTAS Level 2 n = 205 (26.9%) KTAS Level 3 n = 353 (46.3%] KTAS Level 4 n = 204 (26.77%)	Human transcribed and ML-transcribed simulated triage dialogue	BERT	SVM, KNN, RF	Model performance with IBM’s auto-transcribed test dataset. BERT-KTAS AUC 0.82 (0.75–0.87) SVM AUC 0.86 (0.81–0.90) KNN AUC 0.89 (0.85–0.93) RF AUC 0.86 (0.82–0.9)
Ivanov et al. (2021)	Assignment of triage score (ESI).	147 052 patient encounters (age 1 year or older)	ESI 1 n = 693 (0.42%) ESI 2 n = 19 363 (11.65%) ESI 3 n = 82 295 (49.52%] ESI 4 n = 54 596 (32.85%) ESI 5 n = 8644 (5.20%] ESI missing n = 585 (0.35%)	Blood glucose, chief complaint (free-text), demographics, FHx, history of presenting complaint (free-text), mental status, mode of arrival, pain score, SHx, vitals	Clinical-NLP (developed by authors), XGBoost	Nurse triage	Clinical-NLP model AUC 0.85 Nurse triage AUC 0.75 Clinician one AUC 0.86 Clinician two AUC 0.85 Clinician three AUC 0.82
Sterling et al. (2020)	Assignment of triage score (ESI).	226 317 patient encounters (adult and paediatric)	0 resources = 30 604 (13.5%) 1 resource = 49 315 (21.8%) 2 or more resources = 146 398 (64.7%)	Chief complaint (structured), demographics, history of presenting complaint (free-text), medication, mental status, mode of arrival, pain score, PMHx, vitals	LSTM	Emergency nurses (2)	Model predictions (on nursing prediction subset) F1 = 0.589 Accuracy 0.659 Nurse predictions (on 1000 presentations) F1 = 0.592 Accuracy 0.690
Wang et al. (2019)	Assignment of triage score.	70 918 patient encounters (adult)	Not reported	Chief complaint (free-text), demographics, history of presenting complaint (free-text), physical examination (free-text), vitals	“DeepTriager” model (based on LSTM+DNN)	NEWS + LR/BOW/RF	NEWS + LR AUC 0.8631 NEWS + BOW + LR AUC 0.9016 NEWS + BOW + RF AUC 0.9257 NEWS + LSTM AUC 0.9525 “DeepTriager” AUC 0.9594
Choi et al. (2019)	Assignment of triage score (KTAS).	138 022 patient encounters (adults)	KTAS 1 n = 1989 (1.44%) KTAS 2 n = 16 098 (11.66%) KTAS 3 n = 77 720 (56.31%) KTAS 4 n = 36 045 (26.12%) KTAS 5 n = 6170 (4.47%)	Arrival time, chief complaint (structured), demographics, history of presenting complaint (free-text), mental status, mode of arrival, pain location and intensity, vitals	BoW + LR/RF/XGBoost	None	LR (structured data only) AUC = 0.8812 LR (text data only) AUC = 0.8595 LR (structured and text) AUC = 0.9053 RF (structured and text) AUC = 0.9220 XGB (structured and text) AUC = 0.9220
Gligorijevic et al. (2018)	Assignment of triage score (ESI).	338 500 patient encounters	ESI 2 = 20% ESI 3 = 55% ESI 4 = 22% ESI 5 = 4%	Arrival time, chief complaint (free-text), demographics, history of presenting complaint (free-text), medication, mode of arrival, PMHx, vitals	“Deep Attention Model (DAM)” based on LSTM+DNN	LR, ANN, LSTM, CNN, Approximated nurses’ performance.	LR (structured data only) AUC 0.5277 ANN (structured data only) AUC 0.5689 LSTM (structured + text) AUC 0.8523 CNN (structured + text) AUC 0.8609 DAM (text data only) AUC 0.8763 DAM (structured + text) AUC 0.8797 Approximated nurses’ performance: Accuracy 43.6% DAM (structured + text) Accuracy 59.6%
Tahayori et al. (2020)	Patient disposition (admission or discharge).	249 532 patient encounters (adult)	Admission n = 45 839 (19%) Discharge n = 203 693 (81%)	History of presenting complaint (free-text)	BERT	Emergency consultants (5), Bag-of-words	BERT AUC 0.88 Accuracy 0.83 Emergency Consultant Accuracy 0.78 Bag-of-words AUC 0.77 Accuracy 0.72
Roquette et al. (2020)	Patient disposition (admission or discharge).	499 853 patient encounters (paediatric)	Admission rate = 5.76%	Blood glucose, chief complaint (free-text), demographics, history of presenting complaint (free-text), medication, pain score, past investigation requests, PMHx, triage score (MTS), vitals	LSTM	SVM ElasticNet DNN Catboost (structured) XGBoost Catboost (text)	SVM AUC 0.687 ElasticNet AUC 0.840 CatBoost without text features AUC 0.872 DNN AUC 0.877 XGBoost AUC 0.890 CatBoost with text features AUC 0.891
Arnaud et al. (2020)	Patient disposition (admission or discharge).	Approximately 190 000 patient encounters (adult)	Admission rate approximately 35%	Arrival time, bladder volume, blood glucose, breath alcohol, capillary haemoglobin, capillary ketones, demographics, history of presenting complaint (free-text), mode of arrival, pain, PMHx (free-text), triage score, vitals	CNN (textual data) + ANN (structured data)	None	CNN+ANN AUC ≈ 0.83
Sterling et al. (2019)	Patient disposition (admission or discharge).	256 878 patient encounters.	Admission n = 68 092 (26.51%) Discharge n = 188 786 (73.49%)	History of presenting complaint (free-text)	Paragraph vectors/BoW/Topic modelling + ANN	None	Paragraph vector + ANN AUC = 0.737 Bag-of-words + ANN AUC = 0.785 Topic modelling + ANN AUC 0.687
Zhang et al. (2017)	Patient disposition (admission or discharge).	47 200 patient encounters (paediatric and adult)	Admission 6335 (13.42%)	Arrival time, demographics, chief complaint (free-text), mode of arrival, pain score, PMHx, triage score, vitals, whether the visit was related to an injury/poisoning/adverse effect of medical treatment	BoW + PCA + ANN	LR	LR model 1 (text) AUC 0.742 LR model 2 (structured) AUC 0.824 LR model 3 (structured and text) AUC 0.846 ANN model 1 (text) AUC 0.753 ANN model 2 (structured) AUC 0.823 ANN model 3 (structured + text) AUC 0.844
Joseph et al. (2020)	Identification of critical illness (death within 24 hours of arrival, ICU admission from the ED or within 24 hours of ward admission).	445 925 patient encounters (adult)	Critical illness = 60 901 (13.7%)	Demographics, chief complaint (free-text), triage score (ESI), vitals	LSTM+DNN	DNN (structured data only), LR, XGBoost, Abnormal vital sign trigger, ESI.	Abnormal vital sign trigger AUC 0.521 ESI ≤ 2 AUC 0.672 LR AUC 0.804 Structured data only DNN AUC 0.812 XGBoost AUC 0.820 Combined structured and text data LSTM+DNN AUC 0.857
Fernandes et al. (2020) (1)	Identification of critical illness (ICU admission within 24 hours of triage).	Site one 120 649 patient encounters (adult) Site two 235 826 patient encounters (adult)	Site 1 Critical illness n = 3462 (2.8%) Site 2 Critical illness n = 1784 (0.8%)	Blood glucose, chief complaint (structured and free-text), exams prescribed at triage, mental status, mode of arrival, pain score, time of triage, triage score (ESI or MTS), vitals	Term frequency–inverse document frequency (TF-idf) + LR	LR model trained using only triage priorities (ESI or MTS)	Site 1 ESI only LR AUC 0.78 ESI + clinical variables + chief complaint LR AUC 0.92 Site 2 MTS only LR 0.74 MTS + clinical variables + chief complaint LR 0.86
Fernandes et al. (2020) (2)	Identification of critical illness (in-hospital death or cardiopulmonary arrest within 24 hours of triage).	235 826 patient encounters (adult)	Critical illness = 1121 (0.48%)	Blood glucose, chief complaint (free-text), exams prescribed at triage, mental status, mode of arrival, pain scale, time of triage, vitals	Term frequency–inverse document frequency (TF-idf) + LR/RF/XGBoost	LR trained using only triage priorities (ESI).	ESI only LR AUC 0.85 Clinical variables only XGBoost AUC of 0.96 Clinical variables + chief complaint XGBoost AUC 0.96
Zhang et al. (2019)	Use of advanced diagnostic imaging (CT, US, MRI) during ED visit.	139 150 presentations (adult)	Use of advanced diagnostic imaging = 21.9% CT = 16.8% US = 3.6% MRI = 0.4% Multiple types of imaging = 1.2%	Arrival time, demographics, history of presenting complaint (free-text), mode of arrival, pain scale, PMHx, triage score, vitals, whether the visit was related to an injury/poisoning/adverse effect of medical treatment	Latent Dirichlet Allocation (LDA) algorithm + LR	None	Any advanced diagnostic imaging use LDA + LR Unstructured variables AUC 0.74 Structured variables AUC 0.69 Unstructured + Structured AUC 0.78
Zhang et al. (2019)	Performance of any diagnostic imaging during ED visit.	27 665 patient encounters (paediatric)	Any imaging n = 8394 (30.3%) X-ray n = 6922 (4.9%) CT n = 1367 (4.9%)	Arrival time, demographics, history of presenting complaint (free-text), mode of arrival, pain scale, PMHx, triage score, vitals, whether the visit was related to an injury/poisoning/adverse effect of medical treatment	BoW + PCA + LR	None	BoW + PCA + LR Any imaging Unstructured variables AUC 0.810 Structured variables AUC 0.706 Unstructured + structured AUC 0.824
Chang et al. (2020)	Prediction of provider-assigned chief complaint label.	1 799 365 free-text chief complaints (adult and paediatric)	Top 25 chief complaint labels accounted for 50.30% of dataset	History of presenting complaint (free-text)	BERT	LSTM, ELMo	Full dataset (434 labels) BERT Accuracy Top-1 0.65 Top-5 0.92 ELMo Accuracy Top-1 0.63 Top-5 0.90 LSTM Accuracy Top-1 0.63 Top-5 0.90
Greenbaum et al. (2019)	Percent of presenting problems entered at triage able to be automatically mapped to a structured ontology.	279 231 patient encounters total (78 157 patient encounters were post-implementation)	Pre-implementation = 55 users Development period = 85 users Post- implementation = 53 users	Demographics, history of presenting complaint (free-text), pain score, triage score (ESI), vitals	BoW + SVM	Pre-implementation practice	Pre-implementation Structured data capture 26.2% Keystrokes per presenting problem 11.6 Post-implementation Structured data capture 97.2% Keystrokes per presenting problem 0.6 Higher overall quality (qualitative)
Horng et al. (2017)	Diagnosis of infection in the emergency department.	230 936 patient encounters	Diagnosis of infection n = 32 103 (14%)	Demographics, chief complaint (free-text), history of presenting complaint (free-text), pain score, triage score (ESI), vitals	BoW/Topic model + SVM	LR, RF, Naive Bayes	SVM (structured) AUC 0.67 SVM (structured + text) AUC 0.86 LR (structured + text) AUC 0.86 Naïve Bayes (structured + text) AUC 0.83 RF (structured + text) AUC 0.87
Irvine et al. (2008)	Identification and classification of temporal expressions used in triage notes.	598 triage notes	All manually coded temporal expressions = 1041	Arrival time, History of presenting complaint (free-text)	“Triage Note Temporal Information Extraction System (TN-TIES)” based on decision tree classifiers or Naive Bayes	None	TN-TIES (decision tree) Relative Date and Time Positive predictive value = 94% Sensitivity = 86% TN-TIES (Naive Bayes) Reported to not perform as well as decision tree (exact values not provided).

Abbreviations

KTAS—Korean Triage and Acuity Scale

ESI—Emergency Severity Index

ICU—Intensive Care Unit

ED—Emergency Department

ML—Machine learning

FHx—Family history

SHx—Social history

PMHx—Past medical history

Vitals—Respiratory rate (RR), heart rate (HR), systolic blood pressure (SBP), diastolic blood pressure (DBP), temperature (Temp), and oxygen saturation (SPO2).

MTS—Manchester Triage system

BERT—bidirectional encoder representations from transformers

XGBoost—eXtreme Gradient Boosting

LSTM—Long short-term memory

DNN—Deep neural network

LR—Logistic regression

RF—Random forest

CNN—Convolutional neural network

ANN—Artificial neural network

BoW—Bag-of-words

PCA—Principal component analysis

SVM—Support vector machine

KNN—k-nearest neighbors

F1—The harmonic mean of precision (positive predictive value) and recall (sensitivity)

AUC—Area under the receiver operating characteristic curve

ELMo—Embeddings from Language Model

NEWS—National Early Warning Score

### Prediction of critical illness

Of the three studies that predicted critical illness at triage (defined as ICU admission, cardiopulmonary arrest within 24 hours, or death within 24 hours of triage), Fernandes et al. achieved the highest AUC (0.96) in predicting in-hospital death or cardiopulmonary arrest within 24 hours of triage using an extreme gradient boosting model (Table 2) [43–45]. They found no difference in AUC when using clinical variables only or clinical variables and structured chief complaint processed by NLP. Joseph et al. found their NLP model (AUC 0.857) significantly outperformed an abnormal vital sign trigger (AUC 0.521) and ESI ≤ 2 (AUC 0.672) in predicting critical illness. The addition of free-text data improved the performance of their neural network model (from AUC 0.820 to AUC 0.857).

### Prediction of triage score

NLP has been retrospectively applied to data acquired from multiple different triage systems [17, 36–38, 47, 48]. NLP models and multimodal models have achieved high AUCs in assigning triage categories using structured and free-text data (Table 2) [17, 36–38, 47, 48]. Wang et al. achieved the highest performance in predicting ESI using their "DeepTriager" model (AUC 0.96). Kim et al. achieved an AUC of 0.89 in assigning a KTAS category to auto-transcribed simulated triage dialogue. This was only slightly lower than the performance achieved using human-transcribed simulated triage dialogue (AUC 0.90).

Three studies compared the accuracy of triage scores assigned by multimodal models incorporating NLP to triage scores assigned by nurses [17, 36, 47]. Such models were reported to be more accurate than nurses in two out of three papers [17, 36, 47]. Ivanov et al. (2021) used a random sample test set of 729 records to assess their model’s ability to predict ESI. In this test set, ESI had been assigned to each record with unanimous agreement by three expert clinicians. They also compared ED site nurses’ original ESI against the expert consensus assigned ESI. When applied to the test set, their clinical-NLP model achieved an AUC of 0.85 in predicting ESI and original ED nurse triage achieved an AUC of 0.75. Three members of the study team (two emergency clinicians and one emergency nurse) also assigned ESI to this test set, achieving similar AUCs to the NLP model (clinician one AUC 0.86, clinician two AUC 0.85, clinician three AUC 0.82). Sterling et al. (2020) retrospectively calculated number of resources used (ESI), and then used a test set of 1000 randomly selected records to compare their model performance against two experienced ED nurses. In this test set, their model achieved a similar F1 score (0.589 vs 0.659) but lower accuracy (0.589 vs 0.659) than the two ED nurses in predicting number of resources used. Gligorijevic et al. (2018) approximated nurses’ performance by analysing predicted (ESI) versus actual number of resources used. They then compared approximated nurses’ performance against their model’s capability at predicting actual resources used. Overall, their NLP model achieved a higher accuracy than approximated nurses’ performance in assigning number of resources used category (43.6% vs 59.6%). The addition of text data compared to structured data alone improved performance in assigning triage score [36, 37].

### Prediction of provider-assigned chief complaint

NLP models and multimodal models incorporating NLP were able to accurately map free-text history of presenting complaint to structured chief complaints (Table 2) [42, 50]. Chang et al. (2020) used BERT to predict provider-assigned chief complaint labels (Top-5 structured label AUC 0.92). Greenbaum et al. (2019) applied NLP to free text triage notes to rank structured chief complaint labels by their predicted probability. This improved structured data capture from 26.2% to 97.2%.

### Prediction of investigations

Multimodal models incorporating NLP were used to predict diagnostic imaging performed in the ED (Table 2) [39, 40]. Zhang et al. developed a model to predict need for advanced diagnostic imaging (computed tomography, ultrasound, magnetic resonance imaging) in the ED, and obtained an AUC 0.78. Zhang et al. also achieved an AUC 0.824 in predicting the need for any diagnostic imaging in a paediatric population. In both cases, the inclusion of structured variables improved performance slightly when compared to unstructured variables alone (AUC from 0.74 to 0.78, and AUC from 0.81 to 0.82).

### Identifying infection

Horng et al. (2017) found that the incorporation of free-text data improves the discriminatory ability (increase in AUC from 0.67 to 0.86) for identifying sepsis (defined by ICD-9-CM code) in the ED at triage (Table 2).

### Extracting temporal information from triage notes

Irving et al. (2008) applied an NLP system to extract and classify temporal information contained in free-text triage notes. Such information included the relative time of event compared to triage time, and duration of event. They report better performance was obtained using a Decision Tree compared to Naive Bayes.

### Multimodal models

Eleven papers compared ML models that used only structured data to multimodal models that incorporated both structured data and free-text data (Table 2) [34–40, 43–46]. The best performing model in each of these papers incorporated free-text. The largest improvement in model performance from incorporating free-text was found by Horng et al. (increase in AUC from 0.67 to 0.86 for identifying infection). The addition of free-text did not improve model AUC in one case, however, did improve model average precision [44]. There were no cases where the incorporation of free-text into the model resulted in worse performance. Six papers assessed models that used only free-text, with no structured data [30, 36, 37, 39, 40, 42]. Tahayori et al. were able to use only free-text data to predict admission with high accuracy (83%). Zhang et al. used free-text to predict performance of diagnostic imaging. Gligorijevic’s “Deep Attention” models using only unstructured data outperformed those using only structured data. Incorporating both structured data and free-text data improved results when compared to models that used only free-text data, though often only a small improvement was found.

### Modern NLP compared to traditional NLP

Three papers directly compared modern NLP based on DL to more traditional ML techniques such as bag-of-words and topic modelling (Table 2) [30, 38, 48]. Modern DL based NLP outperformed traditional ML based NLP in two cases [30, 38]. In contrast, Kim et al. found that a BERT based DL model did not perform better than ML based models, though their population was relatively small. Chang et al. compared the performance of multiple modern DL based models, finding BERT slightly outperformed Embeddings from Language Models (ELMo) and Long Short-Term Memory (LSTM) networks in mapping free-text chief complaints to structured fields.

### Integration into practice

Greenbaum et al. was the only study that reported the deployment of an NLP based model into clinical practice. Greenbaum et al. aimed to increase the ease of high-quality structured data collection of patients’ chief complaint at triage through the use of an NLP based model. Their model used both free-text triage notes and structured data to provide contextual autocomplete of chief complaint label, and also show the user a list of the top five most likely chief complaints. Prior to implementation of their model, chief complaint was more commonly entered as unstructured free text, with only 26.2% of patient encounters resulted in structured data capture. Following implementation, this increased to 97.2%. The authors aggregated multiple incidents of unscheduled downtime that occurred throughout the study to opportunistically assess the impact of their model. When ML based autocomplete was not operational (and instead alphabetised autocomplete was shown), the percent of encounters that resulted in structured data capture decreased from 97.2% to 89.2%. The number of keystrokes typed for each presenting problem decreased from 11.6 pre-implementation to 0.6 post-implementation. Contextual autocomplete was associated with qualitatively more complete and higher quality (as assessed by three independent reviewers on a four-point Likert scale) structured documentation of chief complaints.

### Study quality—Risk of bias within and across studies

A summary of the PROBAST assessment is provided in Table 3. Overall, 16 out of 20 studies were considered to have a high risk of bias. A common reason for overall high risk of bias in the PROBAST assessment was lack of external validation. Four studies were assessed as having a low risk of bias. One study had high applicability concerns and 19 studies had low applicability concerns. The four studies assessed as having low risk of bias also had low applicability concerns. No studies referred to a previously published or publicly registered protocol.

**Table 3:** PROBAST assessment of the included studies.

Study	Risk of Bias (ROB)				Applicability			Overall	
	Participants	Predictors	Outcome	Analysis	Participants	Predictors	Outcome	ROB	Applicability
Kim et al. 2021	?	?	+	‐	‐	‐	+	‐	‐
Ivanov et al. 2021	+	+	?	‐	+	+	+	‐	+
Tahayori et al. 2020	+	+	+	+	+	+	+	+	+
Sterling et al. 2020	?	+	?	‐	+	+	?	‐	+
Roquette et al. 2020	+	+	+	+	+	+	+	+	+
Joseph et al. 2020	+	+	+	‐	+	+	+	‐	+
Fernandes et al. 2020 (2)	‐	+	?	+	+	+	+	‐	+
Fernandes et al. 2020 (1)	‐	+	?	+	+	+	+	‐	+
Chang et al. 2020	+	+	‐	+	+	+	+	‐	+
Arnaud et al. 2020	‐	+	+	‐	+	+	+	‐	+
Zhang et al. 2019 (2)	?	+	+	‐	+	+	+	‐	+
Zhang et al. 2019 (1)	?	+	+	‐	+	+	+	‐	+
Wang et al. 2019	+	+	+	‐	+	+	+	‐	+
Sterling et al. 2019	+	+	+	‐	+	+	+	‐	+
Greenbaum et al. 2019	+	+	+	+	+	+	+	+	+
Choi et al. 2019	+	+	?	‐	+	+	+	‐	+
Gligorijevic 2018	+	+	‐	‐	+	+	+	‐	+
Zhang et al. 2017	+	+	+	+	+	+	+	+	+
Horng et al. 2017	+	+	‐	+	+	+	+	‐	+
Irvine et al. 2008	?	+	?	‐	+	+	+	‐	+

PROBAST = Prediction model Risk Of Bias ASsessment Tool; ROB = risk of bias.

+ indicates low ROB/low concern regarding applicability

− indicates high ROB/high concern regarding applicability; and

? indicates unclear ROB/unclear concern regarding applicability

### Availability of datasets and code

Availability of study datasets and code is shown in Table 4. Data was publicly available for three studies (all by Zhang et al.) and was available on request from study authors for a further four studies [30, 34, 35, 39, 40, 43, 44]. One study reported plans to release a modified de-identified dataset, however at the time of this review this is still pending approvals [45]. The model code was publicly available for two studies [42, 45]. Notably, the code repository from Chang et al. was well organised and contained clear instructions for researchers on how to download their pretrained model and apply it to their own dataset.

**Table 4:** Availability of dataset and code for included studies.

Study	Dataset available	Code available
Kim et al. 2021	No	No
Ivanov et al. 2021	No	No
Tahayori et al. 2020	Yes*	No
Sterling et al. 2020	No	No
Roquette et al. 2020	No	No
Joseph et al. 2020	No**	Yes
Fernandes et al. 2020 (2)	Yes*	No
Fernandes et al. 2020 (1)	Yes*	No
Chang et al. 2020	No	Yes
Arnaud et al. 2020	No	No
Zhang et al. 2019 (2)	Yes ***	No
Zhang et al. 2019 (1)	Yes ***	No
Wang et al. 2019	No	No
Sterling et al. 2019	No	No
Greenbaum et al. 2019	No	No
Choi et al. 2019	No	No
Gligorijevic 2018	No	No
Zhang et al. 2017	Yes***	No
Horng et al. 2017	Yes*	No
Irvine et al. 2008	No	No

* Data available on request from the authors and may be released to researchers following the signing of a data sharing agreement.

** Pending approval, a modified, de-identified dataset containing modified chief complaint text data will be uploaded. Approval still pending at time of this review.

*** All data freely and publicly available.

## Discussion

### NLP at triage

This review finds that NLP has been applied to data available at the time of ED triage to predict a range of outcomes, with a focus on predicting need for admission and assigned triage score. This review also the combining free-text nursing triage notes with structured data appears to result in the best model performance, however free-text nursing triage notes alone have been used by NLP algorithms to predict need for admission and need for diagnostic imaging [18, 30, 39, 40]. A potential benefit of developing models that require only free-text as an input is that it may allow for easier portability of predictive models between different triage systems [30].

### Structured data capture

Accurate and consistent structured capture of patients’ presenting complaints is important for research, service improvement, and public health initiatives [50]. Common medical ontologies also improve system interoperability [51]. However collection of structured data is often difficult, especially when contrasted with the ease and expressiveness of free-text entry [50]. In a rare singular example of NLP being deployed into routine clinical practice at ED triage, Greenbaum et al. developed, implemented, and prospectively evaluated an NLP driven user interface in an attempt to improve structured data capture [50]. Promisingly, they report that their NLP based contextual auto-predict did not add additional burden to users, made structured data collection easier than unstructured data collection, and significantly increased structured data collection.

### NLP compared to humans

Human performance may be a reasonable baseline for ML models to meet to be considered accurate enough for implementation into clinical practice. Few studies have compared NLP models at triage to human performance. Such comparisons will be crucial in future work. Tahayori et al. was the only study that compared results from NLP models to emergency physicians [30]. Ivanov et al., Sterling et al, and Gligorijevic et al. compared NLP based models to nurses in assigning triage scores and found model accuracy was similar to nurses [17, 36, 47].

### Modern NLP

While it is difficult to compare studies due to their heterogeneity, advanced DL based NLP appears to outperform traditional NLP. This is certainly the case when compared internally within studies and is consistent with previous NLP research [52]. BERT appears to be the most popular advanced NLP that has been used. BERT was released in October 2018 and at the time of release, BERT outperformed other NLP models [27]. However, of the 16 papers published since the release of BERT, only three have used it. Other large models have subsequently been released. For example, GPT-3 is a 175 billion parameter language model that was released in 2020 and is reported to outperform BERT in various circumstances [28]. Chowdhery et al. have recently published Pathways Language Model (PaLM), a 540-billion parameter model that achieves further increases in performance [29].

## Future directions

NLP at ED triage appears to be a promising area for future research. Triage datasets often contain a large volume data with clearly labelled outcomes (such as admission or discharge), which is useful for developing NLP models. Triage information may be available hours before emergency physician documentation, and accurate predictions made at triage have the potential to increase healthcare system efficiency [18]. There is also the possibility of close human oversight if deployed in practice. Future work could aim to predict other important patient-oriented outcomes at the time of triage such as wait times, need for advanced cardiovascular investigations, or need for surgery.

### Incorporating clinical gestalt

Sterling et al. 2020 noted the difficulty in capturing the general clinical impression of the triage nurse [17]. Ivanov et al. also noted that important contextual aspects at triage were not available for consideration by ML models [47]. Future work could assess the impact of incorporating triage nurses’ gestalt into predictive models. Other contextual data available at the time of triage includes the number of patients currently waiting to be seen, the number of patients currently in the ED, and number of admitted patients in the hospital could also be incorporated into ML models. However, it is unknown if the inclusion of such data would improve model performance.

### Integration with other AI systems

Kim et al. provides an interesting example of how various AI based technologies can be combined [48]. Future work could assess if it is feasible to integrate triage NLP models with other novel AI based interventions, such as automated monitoring of patients’ vital signs while they are in the waiting room, or with data entered by patients themselves in self-triage applications. However, combining multiple AI based predictive models may not result in improved performance.

### Pre-trained models for ED triage

Publicly available large DL based language models have often been trained on corpuses containing text from newspapers, books, and websites [27, 28]. Triage notes are often quite short and contain a number of unique and idiosyncratic abbreviations and acronyms not common in everyday English language [17, 30]. NLP models that have been applied to triage notes were often based on models that were not developed specifically for this purpose. DL based NLP models that have been fine-tuned on large corpuses of medical text have been released, however they have not been applied to ED triage. Large publicly available clinical databases such as MIMIC-IV that contain ED triage notes with linked outcomes may be helpful in further model development and may facilitate direct comparisons between models developed by different research groups [53, 54]. Triage focused NLP research could potentially benefit from groups sharing large language models that have been pre-trained on triage data, though it is unknown whether such models’ performance would generalise across different healthcare settings and triage systems.

### Interpretability

NLP models have become increasingly complex. Inputs to DL based NLP models may be processed through billions of interconnected processing units prior to the model generating an output [26]. This has increased their predictive capability, however the explanation of why a model gives a certain output when presented with a particular input has become less interpretable to humans. Some have suggested that it is unethical to implement such models into clinical practice if the reasoning behind their predictions cannot be interpreted by humans [55]. It has also been suggested that improved interpretability may help end users detect model biases and improve patient safety [56]. Other however, argue that model interpretability is unnecessary and should not be pursued at the expense of model accuracy [57]. It is currently unknown if clinicians will accept a model into routine clinical practice if the model cannot explain why an output was given in a way that is interpretable to humans. Developing “explainable AI” models is an active area of research [58, 59]. Few papers attempted to address human interpretability of their NLP model’s output. Wang et al. show how models could be somewhat more interpretable [38]. Their triage model is able to highlight free-text triage notes, with a darker colour corresponding to the sections of text that was more heavily weighted by the model. This provides an initial "sense check" that humans can then combine with their own experience and knowledge.

### Prospective and external validation is needed

The majority of research so far has been retrospective with a high risk of bias, and completed in the USA. There is a significant need for prospective evaluation and external validation, especially in other countries and triage systems. Further research is also required to assess the impact of integrating NLP at triage on patient orientated outcomes, as there is currently little evidence that NLP at triage improves outcomes compared to usual clinical practice.

### Clinical impact and risk

NLP models have rarely been deployed at ED triage. As such, it is unknown what impact these tools could have on clinical practice. The introduction of a new tool into a complex system is likely to have unintended consequences, and use of the tool may itself change practice. Triage notes may be written in a different way if it is known that they are being used for predictive purposes. It is also unknown if the length of triage notes impacts model performance. There may also be unintended harms. For example, telling a patient at triage that they are likely to be admitted or to have a long wait time, could potentially influence their behaviour and affect the number of patients who leave without being seen. It may be useful to establish the performance benchmarks predictive models must meet prior to implementation into clinical practice. This could be achieved through further studies comparing NLP model performance to emergency physicians and nurses.

Predictive models are trained on data that reflects current practice. This engrains the assumption that current practice is appropriate, which may not be the case. If NLP models are retrained and updated as new data becomes available, then model performance may change over time. It will be important to ensure that there is appropriate algorithm stewardship in place prior to clinical use [60].

### Acceptability

It is also unknown if the use of NLP at triage is acceptable to patients and staff. It will be important to involve clinicians, patients, and healthcare consumer groups in the development and governance of any future implementation projects. It will also be important to ensure that these systems do not place further burden on users. Ease of use and perceived clinical impact will likely be important factors for adoption by clinicians.

### Ethical issues

Racial, age, and gender biases at ED triage have been previously reported [61–63]. Concerns over bias in ML models have been well described [64, 65]. The assessment and reduction of bias in ML is an ongoing and active area of research [66]. At its best, NLP at triage could help reduce bias through standardising triage decisions and providing a more objective triage score. However, at its worst NLP at triage could further ingrain existing biases into practice, under the guise of objectivity and hidden in the opacity of abstract algorithms. Patient apprehensions and concerns about the use of AI will also need to be considered. An emerging body of literature suggests that in while in general, patients view AI positively, they do have some concerns with its use in healthcare [67]. These include perceptions that AI will be less accurate than clinicians, that there is a lack of transparency in predictions, and that there are risks to the privacy of their personal healthcare data [68–73]. Further research investigating the impact of NLP based tools on vulnerable and minority populations is warranted.

## Limitations

### Study level

Only one study contained prospectively validated results, and no studies contained results that were externally validated at a separate site. Results reported may not be generalisable to other settings. There was inconsistent reporting of methods and results among studies. The majority of studies (79%) were assessed to have a high risk of bias.

### Review level

Heterogeneity of the included studies precluded meta-analysis which limits the level of evidence this review provides. All studies reported positive results for NLP at triage, which may reflect publication bias. While we took significant care to ensure our search strategy was broad enough to capture all relevant literature, the variety of NLP and ML terminology means that some studies may have been missed. Non-English articles, and articles published prior to 2012 were also excluded from our search. We used the PROBAST tool to assess for risk of bias and applicability concerns for included studies. However, other tools to assess risk of bias do exist and their use may have resulted in different risk of bias assessments.

## Conclusion

NLP has been applied to triage data in attempts to predict important patient-oriented outcomes including need for admission and need for critical care. However, there are few examples of implementation into clinical practice and most research is retrospective and at a high risk of bias. Despite these limitations, NLP at triage appears to be a promising area for future research. Further work is needed to prospectively assess the acceptability and clinical impact of implementing NLP at triage on staff, patients, and the healthcare system, and if there are any added benefits over usual clinical practice.

## Supporting information

S1 Checklist PRISMA 2020 for abstracts checklist. (PDF)

S2 Checklist PRISMA 2020 checklist. (PDF)

S1 File Search strategy. Search strategy for PubMed (MEDLINE), Embase, Cochrane Database of Systematic Reviews, Web of Science, and Scopus.
(DOCX)

S2 File (DOCX)
