# Supplementary material for: Applications of natural language processing at emergency department triage: A narrative review
Source: PLoS One. 2023 Dec 14;18(12):e0279953. doi: 10.1371/journal.pone.0279953 (PMC10721204; doi:10.1371/journal.pone.0279953)
Supplement: S1 File — Search strategy for PubMed (MEDLINE), Embase, Cochrane Database of Systematic Reviews, Web of Science, and Scopus. (DOCX) [file pone.0279953.s003.docx]

**S1 Appendix – Search Strategies**

**Medline**

| 1 | | "natural language processing".tw. | |
| --- | --- | --- | --- |
| 2 | | "nlp".tw. | |
| 3 | | exp Data Mining/ | |
| 4 | | "data mining" .tw. | |
| 5 | | "text mining".tw. | |
| 6 | | exp Artificial Intelligence/ | |
| 7 | | "artificial intelligence".tw. | |
| 8 | | "machine learning*.tw. | |
| 9 | | "deep learning".tw. | |
| 10 | | exp Triage/ | |
| 11 | | "triage".tw. | |
| 12 | | 1 or 2 or 3 or 4 or 5 or 6 or 7 or 8 or 9 | |
| 13 | | 10 or 11 | |
| 14 | | 12 and 13 | |
|  | | EBM Reviews Cochrane Database of Systematic Reviews 2005 to September 15, 2021≥  Embase <1974 to 2021 September 15>  Ovid MEDLINE(R) ALL <1946 to September 15, 2021> | |

**Scopus**

((TITLE-ABS-KEY ( "natural language processing") OR TITLE-ABS-KEY ("nlp") OR TITLE-ABS-KEY ( "data mining") OR TITLE-ABS-KEY ( "text mining") OR TITLE-ABS KEY ( "Artificial Intelligence") OR TITLE-ABS-KEY ("machine learning") OR TITLE-ABS-KEY ( "deep learning"))) AND ("triage"))

**Web of Science**

Query 1

"natural language processing" (All Fields) or "nlp" (All Fields) or "data mining" (All Fields) or "text mining" (All Fields) or "Artificial Intelligence" (All Fields) or "machine learning" (All Fields) or "deep learning" (All Fields)

Query 2

"triage" (All Fields)

Search

(#1) AND #2
