# Supplementary material for: Applications of natural language processing at emergency department triage: A narrative review
Source: PLoS One. 2023 Dec 14;18(12):e0279953. doi: 10.1371/journal.pone.0279953 (PMC10721204; doi:10.1371/journal.pone.0279953)
Supplement: S2 File — (DOCX) [file pone.0279953.s004.docx]

| **Year** | **First Author** | **Country** | **Study design** | **Primary Outcome** | **Sites** | **Population** | **Dataset characteristics** | **Input data** | **NLP/ML Model** | **Comparison** | **Results** |
| --- | --- | --- | --- | --- | --- | --- | --- | --- | --- | --- | --- |
| 2021 | Kim | South Korea | Retrospective | Assignment of triage score (KTAS). | Single-centre  (1 site) | 762 simulated triage cases | KTAS Level 2 n= 205 (26.9%)  KTAS Level 3 n = 353 (46.3%]  KTAS Level 4 n = 204 (26.77%) | Human transcribed and ML-transcribed simulated triage dialogue | BERT | SVM, KNN, RF | Model performance with IBM's auto-transcribed test dataset.   BERT-KTAS AUC 0.82 (0.75–0.87)  SVM AUC 0.86 (0.81–0.90)   KNN AUC 0.89 (0.85–0.93)  RF AUC 0.86 (0.82–0.9) |
| 2021 | Ivanov | USA | Retrospective | Assignment of triage score (ESI). | Multi-centre  (2 sites) | 147 052 patient encounters (age 1 year or older) | ESI 1 n= 693 (0.42%)  ESI 2 n = 19 363 (11.65%)  ESI 3 n = 82 295 (49.52%]  ESI 4 n = 54 596 (32.85%)  ESI 5 n = 8644 (5.20%]  ESI missing n = 585 (0.35%) | Blood glucose, chief complaint (free-text), demographics, FHx, history of presenting complaint (free-text), mental status, mode of arrival, pain score, SHx, vitals | Clinical-NLP (developed by authors), XGBoost | Nurse triage | ML Model AUC 0.85  Nurse triage AUC 0.75 |
| 2020 | Tahayori | Australia | Retrospective | Patient disposition (admission or discharge). | Single-centre  (1 site) | 249 532 patient encounters (adult) | 0 resources = 30 604 (13.5%)  1 resource = 49 315 (21.8%)  2 or more resources = 146 398 (64.7%) | History of presenting complaint (free-text) | BERT | Emergency consultants (5),  Bag-of-words | BERT AUC 0.88 Accuracy 0.83 Emergency Consultant Accuracy 0.78  Bag-of-words AUC 0.77 Accuracy 0.72 |
| 2020 | Sterling | USA | Retrospective | Assignment of triage score (ESI). | Multi-centre  (3 sites) | 226 317 patient encounters (adult and paediatric) | Not reported | Chief complaint (structured), demographics, history of presenting complaint (free-text), medication, mental status, mode of arrival, pain score, PMHx, vitals | LSTM | Emergency nurses (2) | Model predictions (on nursing prediction subset)  F1 = 0.589 Accuracy 0.659 Nurse predictions (on 1000 presentations)  F1 = 0.592 Accuracy 0.690 |
| 2020 | Roquette | Brazil | Retrospective | Patient disposition (admission or discharge). | Single-centre  (1 site) | 499 853 patient encounters (paediatric) | KTAS 1 n = 1989 (1.44%)  KTAS 2 n = 16 098 (11.66%)  KTAS 3 n = 77 720 (56.31%)  KTAS 4 n = 36 045 (26.12%)  KTAS 5 n = 6170 (4.47%) | Blood glucose, chief complaint (free-text), demographics, history of presenting complaint (free-text), medication, pain score, past investigation requests, PMHx, triage score (MTS), vitals | LSTM | SVM  ElasticNet  DNN  Catboost (structured)  XGBoost  Catboost (text) | SVM AUC 0.687 ElasticNet AUC 0.840 CatBoost without text features AUC 0.872 DNN AUC 0.877 XGBoost AUC 0.890 CatBoost with text features AUC 0.891 |
| 2020 | Joseph | USA | Retrospective | Identification of critical illness (death within 24 hours of arrival, ICU admission from the ED or within 24 hours of ward admission). | Single-centre  (1 site) | 445 925 patient encounters (adult) | ESI 2 = 20%  ESI 3 = 55%  ESI 4 = 22%  ESI 5 = 4% | Demographics, chief complaint (free-text), triage score (ESI), vitals | LSTM+DNN | DNN (structured data only), LR, XGBoost,  Abnormal vital sign trigger, ESI score. | Abnormal vital sign trigger AUC 0.521  ESI score ≤ 2 AUC 0.672  LR AUC 0.804  Structured data only  DNN AUC 0.812  XGBoost AUC 0.820  Combined structured and text data  LSTM+DNN AUC 0.857 |
| 2020 (1) | Fernandes | Portugal, USA | Retrospective | Identification of critical illness (ICU admission within 24 hours of triage). | Multi-centre  (2 sites) | Site one  120 649 patient encounters (adult)   Site two  235 826 patient encounters (adult) | Admission n = 45 839 (19%)  Discharge n = 203 693 (81%) | Blood glucose, chief complaint (structured and free-text), exams prescribed at triage, mental status, mode of arrival, pain score, time of triage, triage score (ESI or MTS), vitals | Term frequency–inverse document frequency (TF-idf) + LR | LR model trained using only triage priorities (ESI or MTS) | Site 1  ESI only  LR AUC 0.78  ESI + clinical variables + chief complaint  LR AUC 0.92  Site 2  MTS only  LR 0.74  MTS + clinical variables + chief complaint  LR 0.86 |
| 2020 (2) | Fernandes | Portugal, USA | Retrospective | Identification of critical illness (in-hospital death or cardiopulmonary arrest within 24 hours of triage). | Single-centre  (1 site) | 235 826 patient encounters (adult) | Admission rate = 5.76% | Blood glucose, chief complaint (free-text), exams prescribed at triage, mental status, mode of arrival, pain scale, time of triage, vitals | Term frequency–inverse document frequency (TF-idf) + LR/RF/XGBoost | LR trained using only triage priorities (ESI). | ESI only  LR AUC 0.85  Clinical variables only  XGBoost AUC of 0.96  Clinical variables + chief complaint  XGBoost AUC 0.96 |
| 2020 | Chang | USA | Retrospective | Prediction of provider-assigned chief complaint label. | Multi-centre  (7 sites) | 1 799 365 free-text chief complaints (adult and paediatric) | Admission rate approximately 35% | History of presenting complaint (free-text) | BERT | LSTM, ELMo | Full dataset (434 labels)  BERT Accuracy  Top-1 0.65 Top-5 0.92  ELMo Accuracy  Top-1 0.63 Top-5 0.90  LSTM Accuracy  Top-1 0.63 Top-5 0.90 |
| 2020 | Arnaud | France | Retrospective | Patient disposition (admission or discharge). | Single-centre  (1 site) | Approximately  190 000 patient encounters (adult) | Admission n = 68 092 (26.51%)  Discharge n = 188 786 (73.49%) | Arrival time, bladder volume, blood glucose, breath alcohol, capillary haemoglobin, capillary ketones, demographics, history of presenting complaint (free-text), mode of arrival, pain, PMHx (free-text), triage score, vitals | CNN (textual data) + ANN (structured data) | None | CNN+ANN  AUC ≈ 0.83 |
| 2019 (1) | Zhang | USA | Retrospective | Use of advanced diagnostic imaging (CT, US, MRI) during ED visit. | Multi-centre (300 sites) | 139 150 presentations (adult) | Admission 6335 (13.42%) | Arrival time, demographics, history of presenting complaint (free-text), mode of arrival, pain scale, PMHx, triage score, vitals, whether the visit was related to an injury/poisoning/adverse effect of medical treatment | Latent Dirichlet Allocation (LDA) algorithm + LR | None | Any advanced diagnostic imaging use  LDA + LR  Unstructured variables AUC 0.74  Structured variables AUC 0.69  Unstructured + Structured variables AUC 0.78 |
| 2019 (2) | Zhang | USA | Retrospective | Performance of any diagnostic imaging during ED visit. | Multi-centre (300 sites) | 27 665 patient encounters (paediatric) | Critical illness = 60 901 (13.7%) | Arrival time, demographics, history of presenting complaint (free-text), mode of arrival, pain scale, PMHx, triage score, vitals, whether the visit was related to an injury/poisoning/adverse effect of medical treatment | BoW + PCA + LR | None | BoW + PCA + LR Any imaging  Unstructured variables AUC 0.810  Structured variables AUC 0.706  Unstructured + structured AUC 0.824 |
| 2019 | Wang | China | Retrospective | Assignment of triage score. | Single-centre  (1 site) | 70 918 patient encounters (adult) | Site 1  Critical illness n = 3462 (2.8%)  Site 2  Critical illness n = 1784 (0.8%) | Chief complaint (free-text), demographics, history of presenting complaint (free-text), physical examination (free-text), vitals | “DeepTriager” model (based on LSTM+DNN) | NEWS + LR/BOW/RF | NEWS + LR AUC 0.8631 NEWS + BOW + LR AUC 0.9016 NEWS + BOW + RF AUC 0.9257 NEWS + LSTM AUC 0.9525 “DeepTriager” AUC 0.9594 |
| 2019 | Sterling | USA | Retrospective | Patient disposition (admission or discharge). | Multi-centre  (3 sites) | 256 878 patient encounters. | Critical illness = 1121 (0.48%) | History of presenting complaint (free-text) | Paragraph vectors/BoW/Topic modelling + ANN | None | Paragraph vector + ANN AUC=0.737 Bag-of-words + ANN AUC=0.785 Topic modelling + ANN AUC 0.687 |
| 2019 | Greenbaum | USA | Retrospective then prospective | Percent of presenting problems entered at triage able to be automatically mapped to a structured ontology. | Single-centre  (1 site) | 279 231 patient encounters total (78 157 patient encounters were post-implementation) | Use of advanced diagnostic imaging = 21.9%  CT = 16.8%  US = 3.6%  MRI = 0.4%  Multiple types of imaging = 1.2% | Demographics, history of presenting complaint (free-text), pain score, triage score (ESI), vitals | BoW + SVM | Pre-implementation practice | Pre-implementation  Structured data capture 26.2%  Keystrokes per presenting problem 11.6  Post-implementation  Structured data capture 97.2%  Keystrokes per presenting problem 0.6  Higher overall quality (qualitative) |
| 2019 | Choi | South Korea | Retrospective | Assignment of triage score (KTAS). | Single-centre  (1 site) | 138 022 patient encounters (adults) | Any imaging n = 8394 (30.3%)  X-ray n = 6922 (4.9%)  CT n = 1367 (4.9%) | Arrival time, chief complaint (structured), demographics, history of presenting complaint (free-text), mental status, mode of arrival, pain location and intensity, vitals | BoW + LR/RF/XGBoost | None | LR (structured data only) AUC = 0.8812 LR (text data only) AUC = 0.8595 LR (structured and text) AUC = 0.9053 RF (structured and text) AUC = 0.9220  XGB (structured and text data) AUC = 0.9220 |
| 2018 | Gligorijevic | USA | Retrospective | Assignment of triage score (ESI). | Single-centre  (1 site) | 338 500 patient encounters | Top 25 chief complaint labels accounted for 50.30% of dataset | Arrival time, chief complaint (free-text), demographics, history of presenting complaint (free-text), medication, mode of arrival, PMHx, vitals | “Deep Attention Model (DAM)” based on LSTM+DNN | LR, ANN, LSTM, CNN,  Approximated nurses’ performance. | LR (structured data only) AUC 0.5277 ANN (structured data only) AUC 0.5689  LSTM (structured + text) AUC 0.8523  CNN (structured + text) AUC 0.8609  DAM (text data only) AUC 0.8763  DAM (structured + text) AUC 0.8797  Approximated nurses’ performance: Accuracy 43.6%  DAM (structured + text) Accuracy of 59.6% |
| 2017 | Zhang | USA | Retrospective | Patient disposition (admission or discharge). | Multi-centre (642 sites) | 47 200 patient encounters (paediatric and adult) | Pre-implementation = 55 users  Development period = 85 users  Post- implementation = 53 users | Arrival time, demographics, chief complaint (free-text), mode of arrival, pain score, PMHx, triage score, vitals, whether the visit was related to an injury/poisoning/adverse effect of medical treatment | BoW + PCA + ANN | LR | LR model 1 (text) AUC 0.742  LR model 2 (structured) AUC 0.824  LR model 3 (structured and text) AUC 0.846 ANN model 1 (text) AUC 0.753  ANN model 2 (structured) AUC 0.823  ANN model 3 (structured + text) AUC 0.844 |
| 2017 | Horng | USA | Retrospective | Diagnosis of infection in the emergency department. | Single-centre  (1 site) | 230 936 patient encounters | Diagnosis of infection  n = 32 103 (14%) | Demographics, chief complaint (free-text), history of presenting complaint (free-text), pain score, triage score (ESI), vitals | BoW/Topic model + SVM | LR, RF, Naive Bayes | SVM (structured) AUC 0.67  SVM (structured + text) AUC 0.86  LR (structured + text) AUC 0.86  Naïve Bayes (structured + text) AUC 0.83  RF (structured + text) AUC 0.87 |
| 2008 | Irvine | USA | Retrospective | Identification and classification of temporal expressions used in triage notes | Multi-centre (94 sites) | 598 triage notes | All manually coded temporal expressions = 1041 | Arrival time, History of presenting complaint (free-text) | “Triage Note Temporal Information Extraction System (TN-TIES)” based on decision tree classifiers or Naive Bayes | None | TN-TIES (decision tree)  Relative Date and Time  Positive predictive value = 94%  Sensitivity = 86%  TN-TIES (Naive Bayes) Reported to not perform as well as decision tree (exact values not provided). |

Abbreviations

KTAS - Korean Triage and Acuity Scale

ESI - Emergency Severity Index

ICU - Intensive Care Unit

ED - Emergency Department

ML - Machine learning

FHx - Family history

SHx - Social history

PMHx - Past medical history

Vitals - Respiratory rate (RR), heart rate (HR), systolic blood pressure (SBP), diastolic blood pressure (DBP), temperature (Temp), and oxygen saturation (SPO2).

MTS - Manchester Triage system

BERT - bidirectional encoder representations from transformers

XGBoost - eXtreme Gradient Boosting

LSTM - Long short-term memory

DNN - Deep neural network

LR - Logistic regression

RF - Random forest

CNN - Convolutional neural network

ANN - Artificial neural network

BoW - Bag-of-words

PCA - Principal component analysis

SVM - Support vector machine

KNN - k-nearest neighbors

F1 - the harmonic mean of precision and recall

AUC - Area under the receiver operating characteristic curve

ELMo - Embeddings from Language Model

NEWS - National Early Warning Score
